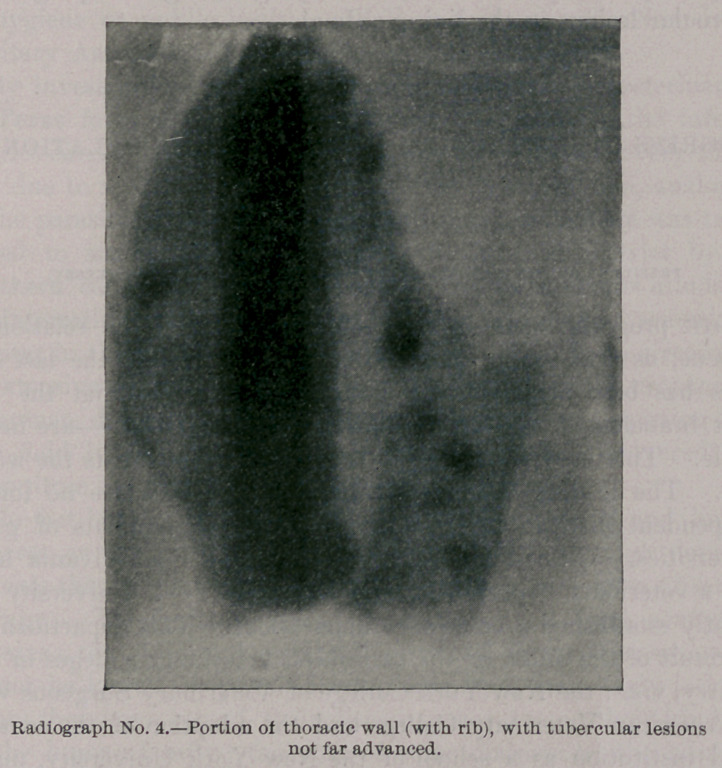# The X-Ray as an Aid in the Diagnosis of Tuberculosis in Cattle1Read before the New Jersey Veterinary Medical Association, at Trenton, January 9, 1902.

**Published:** 1902-02

**Authors:** J. V. Laddey

**Affiliations:** Arlington, N. J.


### THE X-RAY AS AN AID IN THE DIAGNOSIS OF TUBERCULOSIS IN CATTLE.[1]

By J. V. Laddey, D.V.S.,
ARLINGTON, N. J.

The very slow, and in some respects often impractical, way of examining cattle for tuberculosis by means of the tuberculin test has led me to experiment with the X-ray as to the feasibility of detecting the disease in the living animal. I arrived at satisfactory results. The infiltrated calcareous matter, which it seems exists already in the early stages of tubercular lesions, prevents the X-ray from passing through the lesions, thus causing a shadow or opacity. After the successful fluoroscopic test I decided first to have radiographs made, to ascertain whether it would be practical to go to the trouble of experimenting on the living animal. Being compelled to take expense into consideration, I chose specimens not with a view to obtain impressive pictures, but to put the idea to a severe test. Accordingly, I selected lesions with small tubercles in the early stages, as in Plates Nos. 3 and 4, arguing that if disadvantageous specimens showed satisfactory results, the better developed cases would be less penetrable to the rays, and therefore more evident. It is obvious from the photographs that if an enlarged tubercular mediastinal gland were to be radiographed its shadow would be as dense as that thrown by compact bone tissue.

[1] Read before the New Jersey Veterinary Medical Association, at Trenton, January 9, 1902.

Photograph and Radiograph No. 1 present a portion of a pleura spread on cardboard, with tubercular tumors. In the upper left-

Photograph No. 1.—Portion of pleura, with tubercles spread on cardboard.

hand corner is the radiograph of a dime exposed simultaneously with the specimen, to show the comparative opacity of coin and tumors.

Radiograph No. 1.—Portion of pleura, with tubercles spreadon cardboard.

Photograph and Radiograph No. 2 show a tubercular abscess of the lung which is still in the semi-solid, cheesy stage, but which, nevertheless, throws quite a dense shadow.

Photograph and Radiograph No. 3 represent a portion of the diaphragmatic peritoneum, with tubercular lesions in the early stage, when they have a transparent, grayish-pink, rounded, and granular appearance; but even here upon exposure to the rays there is sufficient calcareous matter present to cast a shadow.

Photograph No. 2.—Tubercular abscess of lung.

Photograph and Radiograph No. 4 present a portion of the thoracic wall, with lesions also not far advanced. It also shows the relative density of the shadow cast by the portion of the rib and the tubercular deposits.

The radiographs showing such satisfactory results, I proceeded to make arrangements for a fluoroscopic examination on the living animal. The examination was made in a dark booth erected for the purpose, with a twelve-plate static machine to generate the electrical current. The examination was made on both sides of the animal. An assistant manipulated the "Crookes tube" on one side of the animal, so as to have it directly opposite the fluoroscope through which the cow was examined on the opposite side.

For the first examination the subjects were four thin cows, which were selected because they were thought to be of a tubercular diathesis. The observations through the fluoroscope were as follows :

Cow No. 1. The fluoroscope view on both sides showed a few undefined opacities in the posterior portion of the thorax.

Radiograph No. 2.—Tubercular abscess of lung (cheesy stage).

Cow No. 2. Here the fluoroscopic view presented more and somewhat smaller, but less sharply defined, shadows distributed over the entire thorax.

Cow No. 3. This presented a similar view, with an exceptionally dark spot in region of liver.

Cow No. 4. Showed a clean, unobstructed view, so clear that it enabled me to see the heart in action very distinctly.

My conclusions were that Cows Nos. 1, 2, and 3 would prove to be tuberculous and Cow No. 4 free from tuberculosis. The subjects were then killed and inspected by myself and another inspector of the Bureau of Animal Industry who had purposely not been informed of my conclusions. The post-mortem report is as follows :

In Cow No. 1 the mediastinal glands and the posterior portion of the caudal lobe of the lungs showed tubercular areas.

In Cow No. 2 there existed generalized tuberculosis; there were tubercular lesions in the lungs, liver, and over the entire pleura, but the lesions were small.

Photograph No. 3.—Portion of diaphragmatic peritoneum, with tubercular lesions.

Cow No. 3 showed tuberculosis of lungs and liver. In this case the lesions existed to the largest extent, and especially so in the liver.

Cow No. 4 was entirely free from tuberculosis.

For a second experiment the subjects numbered three. They were good cows, in good condition, supposed to be healthy, considerably fatter than those of the first experiment. Here I observed that adipose tissue does not decrease the penetrability of the rays. Upon fluoroscopic examination I judged Cows Nos. 2 and 3 to be free from tuberculosis, but in Cow No. 1, I noticed a faint

Radiograph No 3.—Portion of diaphragmatic peritoneum, with tubercular lesions.

Photograph No. 4.—Portion of thoracic wall (with rib), with tubercular lesions in the early stage.

opacity near the posterior extremity of the left caudal lobe, and concluded that there might be a slight tubercular deposit in that region, although the shadow was not so pronounced as in those cases of the first test which were found to be tuberculous. Upon post-mortem examination Cows Nos. 2 and 3 were found to be free from tuberculosis; and Cow No. 1 showed, in the very spot where I had detected the shadow, instead of tubercular deposits, lesions of a chronic, circumscribed pleuritic inflammation, with adhesions. The fact that even an indurated serous membrane throws a shadow

Radiograph No. 4.—Portion of thoracic wall (with rib), with tubercular lesions not far advanced.

somewhat different from shadows of other anatomical parts demonstrates the possibilities that might be attained by this method. This latter test taught me also that fluoroscopy is an art of no small importance, in which one can only become proficient through practice. I noticed in this test that I was much better able to discern the different shadows, as of the heart, ribs, liver, etc., than in the previous one; and although a case of tuberculosis in its earliest stages, where there is not a sufficiently appreciable calcareous infiltration present, might escape detection by examination, with the X-ray in the living animal an advanced case could not escape detection.

As this mode of diagnosis consumes only from two to four minutes for an animal, at the utmost, it would recommend itself not only for ordinary diagnostic purposes, but particularly in antemortem work for meat-inspection purposes. It would also be of great value in diagnosing cases of tuberculosis far advanced where the tuberculin test has failed to cause reaction. Furthermore, it could be put to practical use in studying the age and progress of tubercular lesions in the living animal.

## Figures and Tables

**Photograph No. 1. f1:**
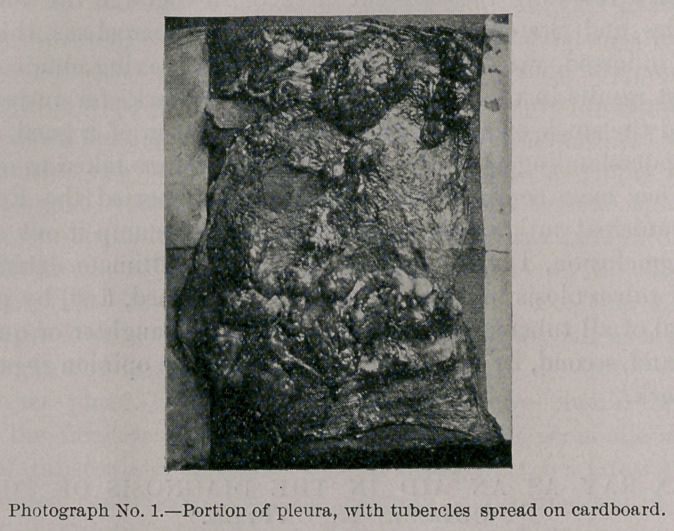


**Radiograph No. 1. f2:**
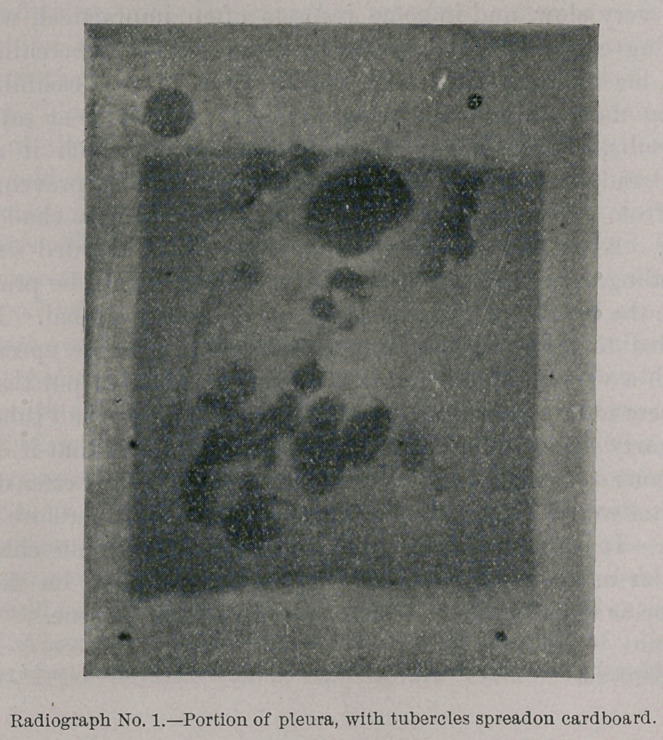


**Photograph No. 2. f3:**
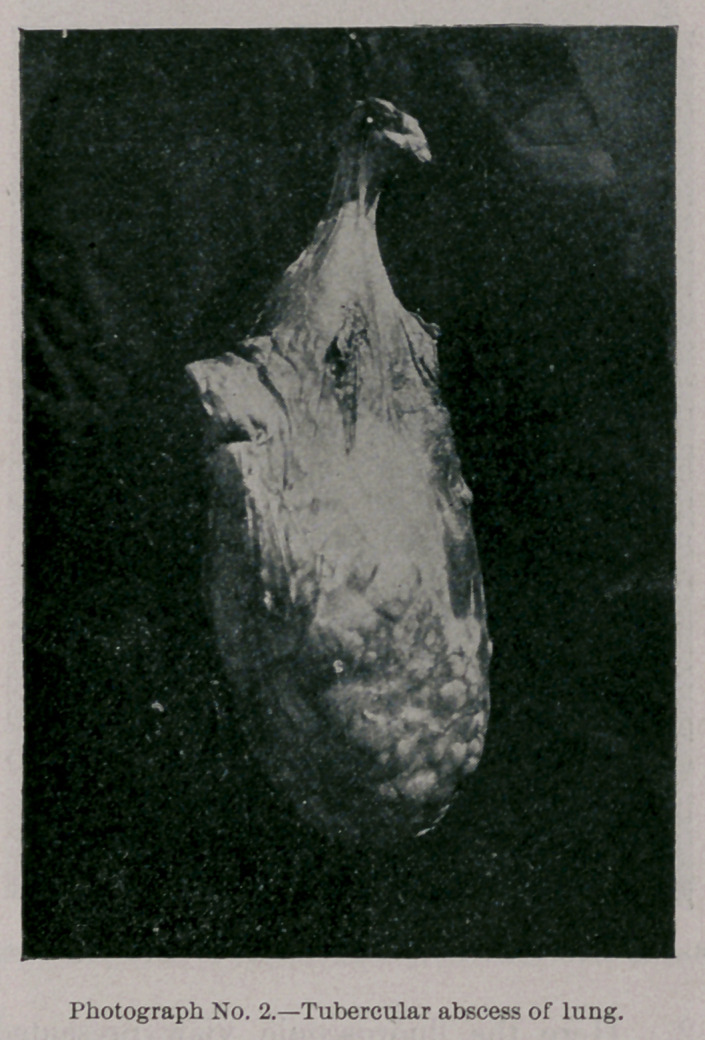


**Radiograph No. 2. f4:**
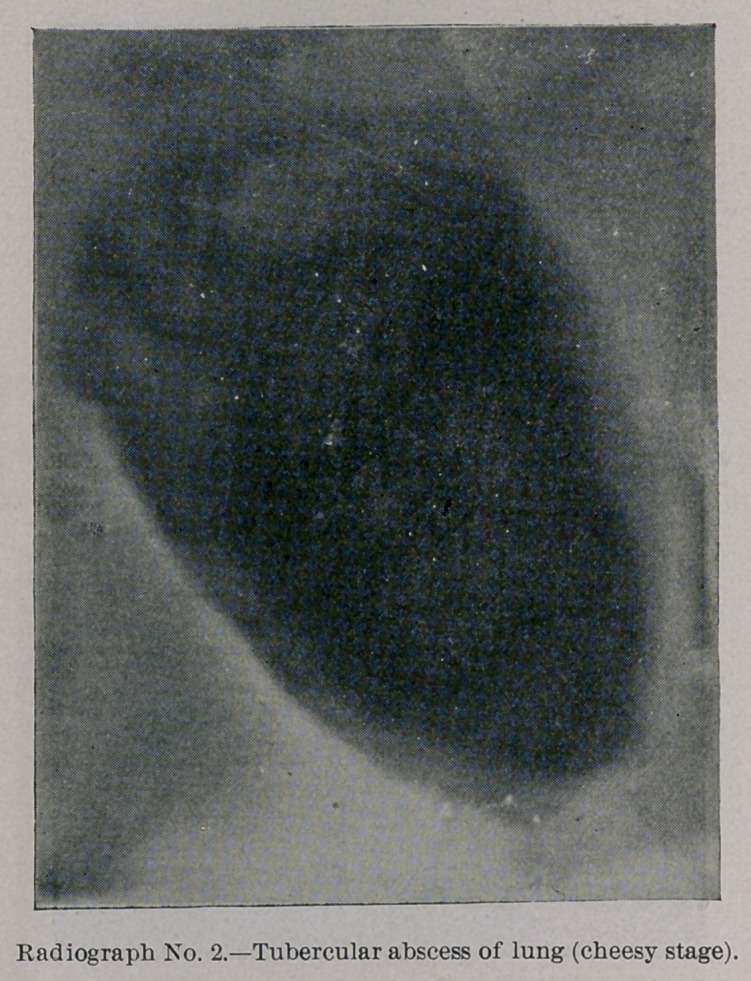


**Photograph No. 3. f5:**
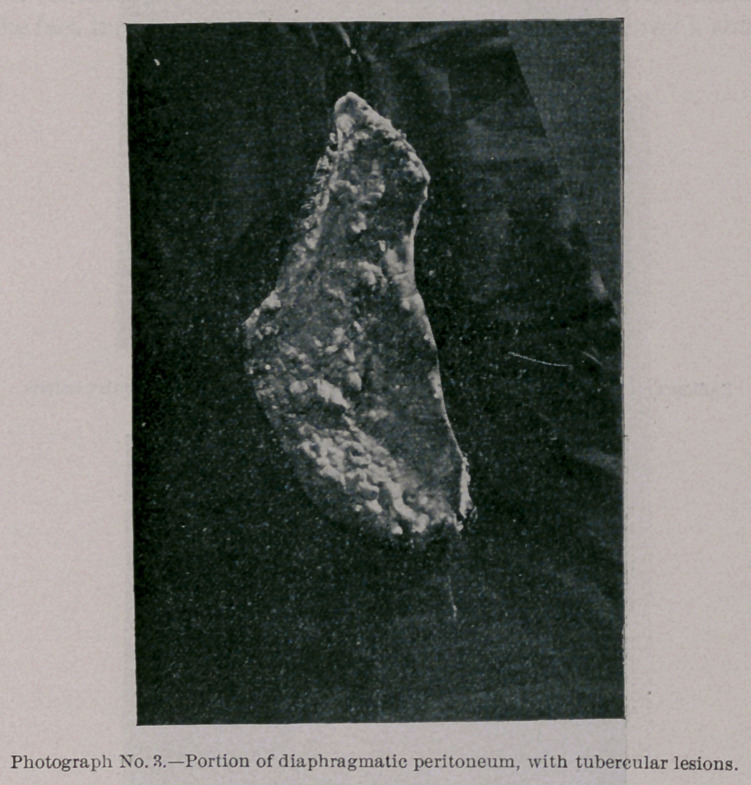


**Radiograph No. 3. f6:**
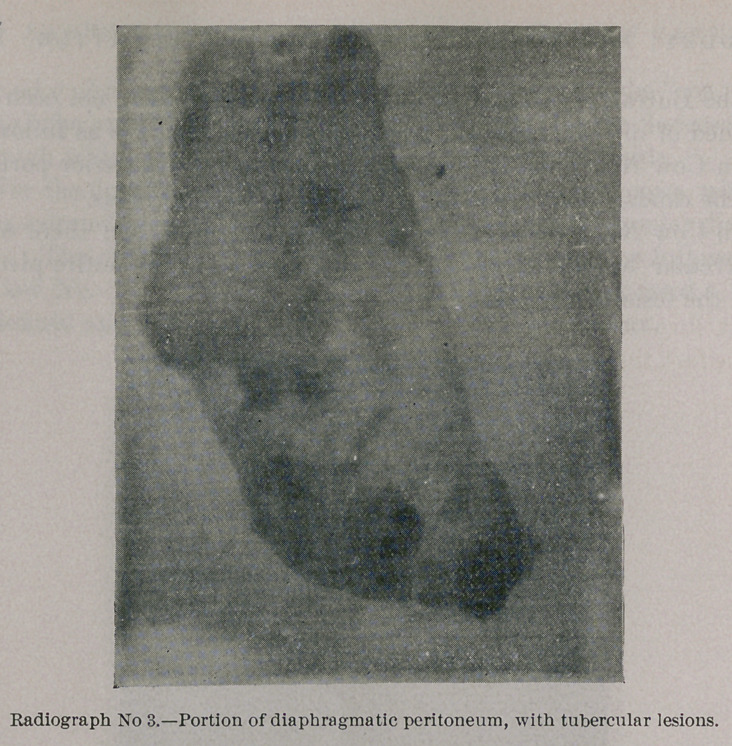


**Photograph No. 4. f7:**
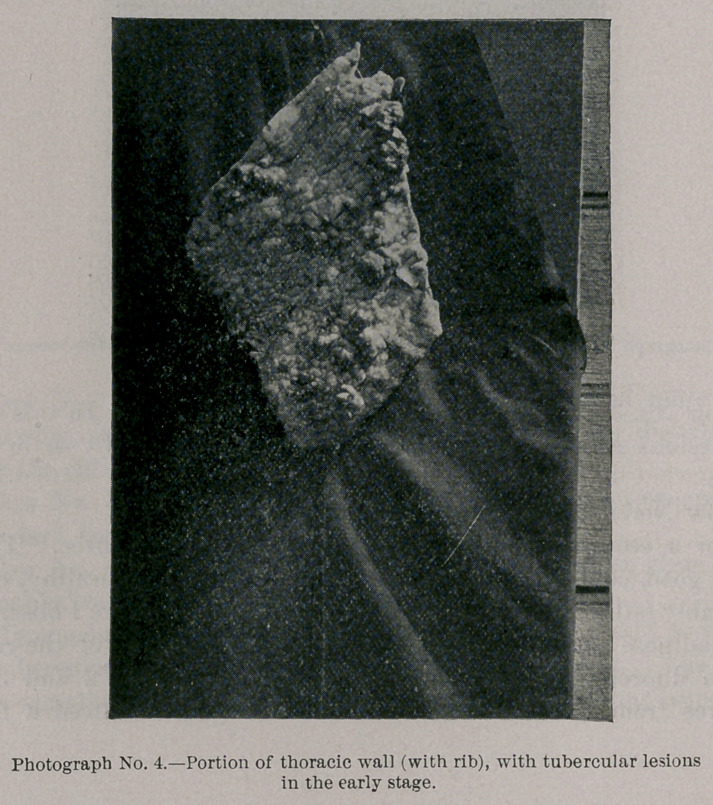


**Radiograph No. 4. f8:**